# Using machine learning for crop yield prediction in the past or the future

**DOI:** 10.3389/fpls.2023.1128388

**Published:** 2023-03-30

**Authors:** Alejandro Morales, Francisco J. Villalobos

**Affiliations:** ^1^ Centre for Crop Systems Analysis, Plant Sciences Group, Wageningen University & Research, Wageningen, Netherlands; ^2^ Instituto de Agricultura Sostenible, Consejo Superior de Investigaciones Científicas (IAS-CSIC), Córdoba, Spain; ^3^ Departamento de Agronomia, ETSIAM, Universidad de Córdoba, Córdoba, Spain

**Keywords:** machine learning, crop simulation model, wheat, sunflower, DSSAT, neural network

## Abstract

The use of ML in agronomy has been increasing exponentially since the start of the century, including data-driven predictions of crop yields from farm-level information on soil, climate and management. However, little is known about the effect of data partitioning schemes on the actual performance of the models, in special when they are built for yield forecast. In this study, we explore the effect of the choice of predictive algorithm, amount of data, and data partitioning strategies on predictive performance, using synthetic datasets from biophysical crop models. We simulated sunflower and wheat data using *OilcropSun* and *Ceres-Wheat* from *DSSAT* for the period 2001-2020 in 5 areas of Spain. Simulations were performed in farms differing in soil depth and management. The data set of farm simulated yields was analyzed with different algorithms (regularized linear models, random forest, artificial neural networks) as a function of seasonal weather, management, and soil. The analysis was performed with Keras for neural networks and R packages for all other algorithms. Data partitioning for training and testing was performed with ordered data (i.e., older data for training, newest data for testing) in order to compare the different algorithms in their ability to predict yields in the future by extrapolating from past data. The Random Forest algorithm had a better performance (Root Mean Square Error 35-38%) than artificial neural networks (37-141%) and regularized linear models (64-65%) and was easier to execute. However, even the best models showed a limited advantage over the predictions of a sensible baseline (average yield of the farm in the training set) which showed RMSE of 42%. Errors in seasonal weather forecasting were not taken into account, so real-world performance is expected to be even closer to the baseline. Application of AI algorithms for yield prediction should always include a comparison with the best guess to evaluate if the additional cost of data required for the model compensates for the increase in predictive power. Random partitioning of data for training and validation should be avoided in models for yield forecasting. Crop models validated for the region and cultivars of interest may be used before actual data collection to establish the potential advantage as illustrated in this study.

## 1 Introduction

Turing (1950) was the first to launch the idea of “learning machines”, applied to digital computers, although the mathematics of artificial neural networks had already been established (McCulloch and Pitts, 1943). Strictly speaking, Machine Learning (ML) is a set of techniques to develop systems able to learn and adapt with minimal or no human intervention. In a broader sense, it encompasses different mathematical techniques used for building data-driven models for prediction and decision-making and can deal with both qualitative and quantitative data. In its infancy, ML, as part of Artificial Intelligence (AI), tried to solve identification problems, which led to the development of Artificial Neural Networks (ANN). Later, in the 1980s, ANN (Dreyfus, 1990) and decision trees (Breiman et al., 1984) became available for regression problems. These algorithms have improved the capacity for generating value from data and has led to a wide variety of algorithms that have thrived in the literature of the past 30 years.

The use of ML in agronomy has been increasing exponentially since the start of the century. In particular, artificial neural networks (ANN) have been used successfully for identification and classification problems that are typical of crop protection (e.g. Mohanty et al., 2016), mechanization of harvest (Bargoti and Underwood, 2017) and product quality sorting (Noh and Lu, 2007). In these cases, no other alternative method exists except for human intervention, so these applications are faithful to the original goals of ML and AI methods. ANN have been also widely used in regression problems, i.e. for fitting empirical quantitative models. They are a powerful alternative to Linear Models (LM) because of their flexibility, i.e. a single hidden layer ANN with enough cells is able to fit any continuous mathematical function within a given interval (Hecht-Nielsen, 1987; Hornik et al., 1989), as long as enough data and computational power are available. General rules have been proposed to determine the data requirement to train ANN’s, in special in classification problems (e.g. Mohanty et al., 2016) but much less is known in the case of regression models for crop yield.

Among the numerous applications of ML in agronomy for regression problems, we find mostly studies directed at calibrating (training according to ML terminology) and testing empirical models for predicting important agronomic variables from proxy measurements. These may be structural canopy parameters (e.g. *LAI*, Kira et al., 2016; biomass, Wang et al., 2019) based on remote data or agronomic variables (yield, crop quality), using soil, weather and management data as inputs (Kaul et al., 2005). In that last application, ML would be an alternative to biophysical crop models (Basso and Liu, 2019) which have the added advantage of providing a framework for understanding the system. Such interpretability is also important in the use of predictions for decision making, since eventually “all models are wrong”. Although frequently claimed in various fields (e.g. Breiman, 2001a), the capacity of ML for revealing the hidden aspects of complex systems in crop production remains to be proven. Additionally, ML algorithms may require larger amounts of data to avoid overfitting (that is the price of their flexibility).

In this paper, we will focus on the application of popular ML algorithms to yield prediction, which is critical for planning farm management and the organization of agriculture-related sectors, like transformation industry, production of inputs, market perspectives, etc. We use “prediction” in both the statistical sense (estimating a dependent variable as a function of a set of independent variables) and in the mathematical sense (forecasting of future events from past data). This distinction is important as the choice of the independent dataset for testing depends on whether the predictive goal is a) to interpolate or infer something in a well-defined system *ex post facto* or b) to extrapolate from past observations on a system into the future (even when the system is not stationary).

We will restrict our analysis to cases where input data do not include remotely sensed variables, which is an ample class per se (e.g. Johnson et al., 2016). In most studies, soil, aggregated weather, management data and yield are used for training and testing ML algorithms. These studies tackle different scales from field (Cao et al., 2021) to country (Hoffman et al., 2018) and different techniques (ANN becoming a common choice).

Our starting hypothesis is that the ability of ML algorithms for yield simulation is much higher for the past (statistical prediction) than for the future (forecasting). A corollary of this would be that the validation of ML should be more restrictive in forecasting.

We used two biophysical crop models, *Ceres-Wheat* (Ritchie et al., 1985) and *OilcropSun* (Villalobos et al., 1996), included in *DSSAT 4.8* (Jones et al., 2003), to generate a vast synthetic data set with absolute control of the sources of variation. The models have been calibrated and validated in different areas, including Southern Spain (Villalobos et al., 1996; Iglesias, 2006). They include the response of crops to nitrogen and water, which are the main limiting factors in their cultivation (Villalobos and Fereres, 2016).

The objectives of this work were a) to analyse the capability of ML methods for yield prediction in sunflower and wheat using a synthetic dataset obtained with crop simulation models and b) to establish guidelines for fair use of ML in crop yield prediction.

## 2 Materials and methods

### 2.1 In silico experiments

The system studied was a set of farms located in 5 regions in Spain (**Table 1**). These regions corresponded to latitudes between 37.5 ° N and 40° N and were distributed from the West (Lobon) to the East (Castellon) of Spain. They are all characterized by a Mediterranean climate with average annual rainfall ranging from 369 mm (Baza) to 611 mm (Cordoba) with high interannual variability (coefficient of variation, CV, between 0.26 and 0.34, see **Table 1**). Reference evapotranspiration and rainfall data are provided in **Supplementary Tables S1** and **S2**.

**Table 1:** Locations representing the centre of each region of the study. Statistics of rainfall refer to the period 2001-2020.

Station	Latitude	Longitude	Altitude	Mean rainfall	SD rainfall
	°	°	m	mm	mm
Lobon	38.85	-6.67	185	436	145
Belmez	38.25	-5.2	503	485	167
Cordoba	37.85	-4.8	80	611	159
Baza	37.57	-2.77	310	369	122
Castellon	39.98	0.03	30	479	130

Each region had a different soil type that was applied to every farm in each region (**Table 2**). Soil depth within each farm vary across three discrete values (different per region) but their contribution to the total area was randomly assigned (**Table 2**). For example, in Lobon, we had one farm with soil depth 1.25 m in 10% of the area, 1.50 m in 60% and 1.75 m in the remainder.

**Table 2:** Soils and farm characteristics of the different regions.

Region	Soil type	Soil depth	#farms	Mean farm size	Range farm size
		m		ha	ha
Lobon	Sandy loam	1.25,1.50,1.75	15	20	15-25
Belmez	Silt loam	0.75,1.0,1.25	12	30	25-35
Cordoba	Clay loam	0.5,1,1.5	11	40	35-45
Baza	Silt loam	0.5,0.75,1.0	12	25	20-30
Castellon	Sandy loam	1.25,1.50,1.75	15	20	15-25

In addition, each farm had a particular combination of cultivar, sowing date, N fertilizer amount and supplementary irrigation. The number of combinations (farms) was randomly assigned to 15 farms in Lobon, 12 in Belmez, Baza and Castellon and 11 in Cordoba (**Table S3**).

Two different set of simulations were run corresponding to monoculture of either sunflower or wheat. This would represent that each farm would devote 50% of the area to each crop as a monocrop. We chose this unrealistic plan in terms of agronomy to separate completely the influence of each crop model on the results.

We simulated sunflower yields with *OilcropSun* (Villalobos et al., 1996) and wheat yields with *Ceres-Wheat* (Ritchie et al., 1985), both included in *DSSAT 4.8* (Jones et al., 2003). The simulations started in 2000 and ended in 2020, in sequence mode, i.e. the N and water balance were continuous for the whole simulation period. All year numbers reported in the following sections refer to the year of harvest. Minimum tillage was applied for both experiments and all the crop residues were left in the field. A change in cultivar occurred at a year uniformly distributed from 2008 to 2012, for the different farms. In sunflower the cultivar was changed from AE353 (moderately short season) to SW-101 (very short, dwarf). The change in cultivar carried a change in planting density from 8 to 16 plants/m^2^ in accordance to the work of Villalobos et al. (1994). For wheat, the initial cultivar was Yecora, a Spring wheat type, which was changed then to a similar cycle cultivar with a 20% higher yield potential. The genetic coefficients of the cultivars are shown in **Table 3**.

**Table 3:** Genetic coefficients of the sunflower and wheat cultivars.

Crop	Cultivar	P1	P2	P5	G2	G3		
Sunflower	AE353	245	3.74	600	1500	3.35		
	SW-101	210	3.74	660	520	2.69		

		P1V	P1D	P5	G1	G2	G3	PHINT
Wheat	SpringW1	5	40	600	20	20	1	100
	SpringW1+	5	40	600	25	20	1	100

P1: Duration of juvenile phase (°C day with base temperature 4°C). P2: Photoperiod response coefficient (days/hour). P5 (for sunflower): Duration of the first anthesis-physiological maturity stage (°C day with base temperature 4°C). G2 (sunflower): Maximum possible number of grains per head. G3 (sunflower): Potential kernel growth rate during the linear kernel filling phase (mg/day). P1V: Vernalization requirement at optimum temperature (days). P1D: Photoperiod response (% reduction in rate/10 h drop in pp). P5 (wheat): Grain filling (excluding lag) phase duration (°C day). G1: Kernel number per unit canopy weight at anthesis (#/g). G2 (wheat): Standard kernel size under optimum conditions (mg). G3 (wheat): Standard, non-stressed mature tiller weight (including grain) (g dwt). PHINT: Interval between successive leaf tip appearances (°C day).

Sowing dates (**Table 4**) were randomly assigned to farms by choosing from 3 alternative dates that were the same in each farm for the 20 years. In those locations where irrigation was feasible (Lobon, Belmez, Castellon), supplementary irrigation was implemented consisting on either zero, one or two 50 mm applications (**Table 4**). The amounts of N were also randomly assigned (**Table 4**) between 75 and 125 kg N/ha, except for Castellon (25-75 kg/ha). The list of farms and their main characteristics are shown in **Table S3**.

**Table 4:** Sowing dates, nitrogen application and irrigation assumed for the different regions where virtual farms were located.

Region	Wheat sowing	Sunflower sowing	N applied	Irrigation
	DOY	DOY	kg/ha	mm
Lobon	4/11, 21/11, 5/12	15/3, 1/4, 15/4	75, 100, 125	50, 100
Belmez	18/10, 4/11, 18/11	15/3, 1/4, 15/4	75, 100, 125	0, 50
Cordoba	14/11, 29/11, 16/12	1/3, 15/3, 1/4	75, 100, 125	0
Baza	16/10, 30/10, 15/11	1/4, 15/4, 1/5	75, 100, 125	0
Castellon	16/11, 30/11, 16/12	15/3, 1/4, 15/4	25, 50, 75	50

### 2.2 Feature selection

After some exploratory analysis a set of 11 input variables (features according to ML terminology) were determined: sowing date (SD), N applied (NA), irrigation applied (IA), anthesis date (AD), rainfall in the preceding fallow and during the growing season (R), mean maximum (TX) and minimum temperature (TN), mean solar radiation (SR), average soil depth in the farm (SD), year (Y) and cultivar (C). The variable to be predicted was the average grain yield for each combination of farm, crop and year. Data was standardized (i.e., subtract mean and divide by standard deviation) using the mean and standard deviations of each variable in the training subset.

It was assumed that the seasonal weather, rainfall and anthesis date in the testing dataset were known exactly (though in real world application these would have to be predicted too, reducing the overall predictive power of the procedure). This decision was taken to focus the analysis exclusively on predicting crop yields from farm- and seasonal-level data, rather than the effect of different weather and phenology forecast methods on yield prediction.

### 2.3 Predictive algorithms

#### 2.3.1 Average yield

The average yield for each cultivar at either the farm or regional level in the training dataset set was used as baselines for predictions. It was the most sensible baseline to compare other algorithms to as it did not make use of any features from the farm that may be correlated with yield. If an algorithm performs worse than this approach, it is considered to be significantly overfitting the data.

#### 2.3.2 Linear model

The linear model included the effects of each feature plus the interactions between cultivars and N, TX, TN, R and SD (i.e. the slopes between these seasonal weather and soil variables and yield were assumed to vary across cultivars). This led to 17 coefficients (2 for average yield per cultivar, 15 for the slopes with respect to year and management, seasonal weather and soil properties, with 5 of these differing between cultivars).

Firstly, the linear model was fitted to all the training data by minimizing the residual sum of squares (RSS):


(1) $$RSS={\sum_{i=1}^{n}\left(y_{i}-\sum_{j=1}^{p}\beta_{j}x_{ij}\right)}^{2},$$

where $y_{i}$ are the values of grain yield, $x_{ij}$ are the values of the different features (including interactions between cultivars and other features) and $\beta_{j}$ are the coefficients in the model.

One may use different techniques to reduce the complexity of the model and avoid overfitting. For example, one may drop coefficients of the model through stepwise regression. A weakness of this approach is that the result may depend on the order in which the model is built (e.g., whether one adds terms or removes terms) and the criterion to determine whether a term should be added or removed. A more general approach is to fit regularized versions of the linear model (also known as shrinkage methods). In a regularized model, a penalty that is a function of the L1 or L2 norm of the coefficients is added to RSS . For the L1 penalty (*Lasso*) we minimized:


(2) $$RSS+P_{L1}=RSS-\lambda \sum_{j=1}^{p}\left|\beta_{j}\right|,$$

where λ is a hyperparameter determined by 30-fold cross-validation using predictive mean absolute error (MAE) as metric. After optimizing the hyperparameter, the resulting model was trained on the entire training dataset. For the L2 norm penalty (*Ridge*) we minimized:


(3) $$RSS+P_{L2}=RSS-\lambda \sum_{j=1}^{p}\beta_{j}^{2}.$$

In the case of the *Lasso* linear model, all coefficients shrink to exactly 0 for a sufficiently high λ , but the goal is to select an optimal λ that produces a sparse linear model where only some of the coefficients are exactly zero. For the *Ridge* linear model, the shrinkage moves asymptotically towards zero as λ grows (but never reaches exactly zero).

The full linear model was fitted with the default linear modelling function in the *stats* R package, whereas the *Lasso* and *Ridge* linear models were fitted with the *glmnet* R package.

#### 2.3.3 Random forest

A random forest (Breiman, 2001b) with a thousand decision trees was trained using the entire training dataset. Default hyperparameters values as provided by the R package *ranger* were used. Each decision tree was fitted on a random sample (with replacement) of the original training dataset. Up to three randomly-selected features were considered for each node split (i.e., the *mtry* hyperparameter was 3) and trees were grown to have leaf nodes with 5 data points (or less) each.

#### 2.3.4 Neural networks

Several artificial neural networks were trained on the entire training dataset with the *Keras* framework using mean square error (MSE) as cost function. Each neural network had one hidden dense layer with a rectified linear activation function, but varied in the number of nodes in the layer from 2 to 12. The total number of trainable parameters (i.e., weights and biases of the hidden and output layer) varied from 29 (2 nodes in the hidden layer) to 169 (12 nodes). The same neural networks were trained a second time but introducing a 50% dropout layer (Srivastava et al., 2014) after the hidden layer, a popular technique designed to reduce overfitting that does not require monitoring a validation set during training.

The training was performed with the Adam algorithm (Kingma and Ba, 2014) with an initial learning rate of 10^−3^. The cost function was monitored throughout the training in order to reduce the learning rate (drop by 2/3 if the cost had not decreased by more than 10^−4^ in 100 epochs) and stop the training (same criterion but with a minimum drop of 10^−6^). For reference, the MSE for standardized yield values was in the order of 0.05 to 0.25. These stopping criteria ensured that the optimization algorithm had converged to a minimum (though no guarantees can be provided that it was the global minimum).

Given the stochasticity inherent to training neural networks, as well as the possibility of multiple local minima in the cost function, the training was repeated five times for each network (each time initializing the weights randomly) and the result with the lowest training cost was chosen.

### 2.4 Evaluation of algorithms

A qualitative test of simulated yields was performed by comparison with observed average values from 2001 to 2020 in the provinces of Cordoba (that includes Belmez and Cordoba) and Granada, where Baza is located. The data were taken from the official statistics of the regional government (Junta de Andalucia, 2022). Such a comparison was not intended as a validation of the crop models but rather to verify that the predicted yields were reasonable.

The evaluation of the predictive power of each algorithm included the following metrics: Root Mean Square Error (RMSE), Mean Absolute Error (MAE), Bias Error (Bias), Maximum Error (ME) and Coefficient of Determination (R2). The values of RMSE, MAE, Bias and ME were divided by the average simulated yield in the corresponding data partition (training or testing) and expressed in percentage.

We also computed the Akaike Information Criterion corrected for small samples (*AICc*) in the training dataset as a popular estimate of predictive performance in the absence of an independent dataset for testing. *AICc* was computed as:


(4) $$\begin{array}{c} AICc=2NLL+2k+\frac{2k^{2}+2k}{n-k-1} \end{array}$$

where NLL is the negative log-likelihood of the data under the trained model (evaluated on the training dataset), k is the number of non-zero parameters in the model and n is the number of data points used for training. The log-likelihood in all cases was calculated assuming that residuals followed a Normal distribution:


(5) $$\begin{array}{c} NLL=\frac{n}{2}\log\left(2\pi \sigma^{2}\right)+\frac{RSS}{2\sigma^{2}} \end{array}$$

where σ is the standard deviation of the residuals and n is the number of data points used to train the algorithm. In the case of linear models and ANN it is clear what the number of parameters are. However, RF is a non-parametric method and therefore there are no parameters being fitted to the data. As an estimate of k we have used the average number of node splits per decision tree inside the random forest, though the real number of degrees of freedom for a random forest is a more complex measure (Mentch and Zhou, 2020).

The process of training and testing was performed for data partitions of 15/5, 10/5 and 5/5 years, where the testing was always performed in the latter years of the simulations (2016-2020). In addition, we performed a random partition over the whole dataset by using 10 repetitions of random data splits (75/25 for training/testing, comparable in amount of training to the 15/5 split). This last data partition exemplifies the testing of algorithms that are meant for interpolation, whereas the former data partitions address the scenario of future yield forecasting with different lengths of past time series.

## 3 Results

### 3.1 Wheat yield

**Figure 1:**
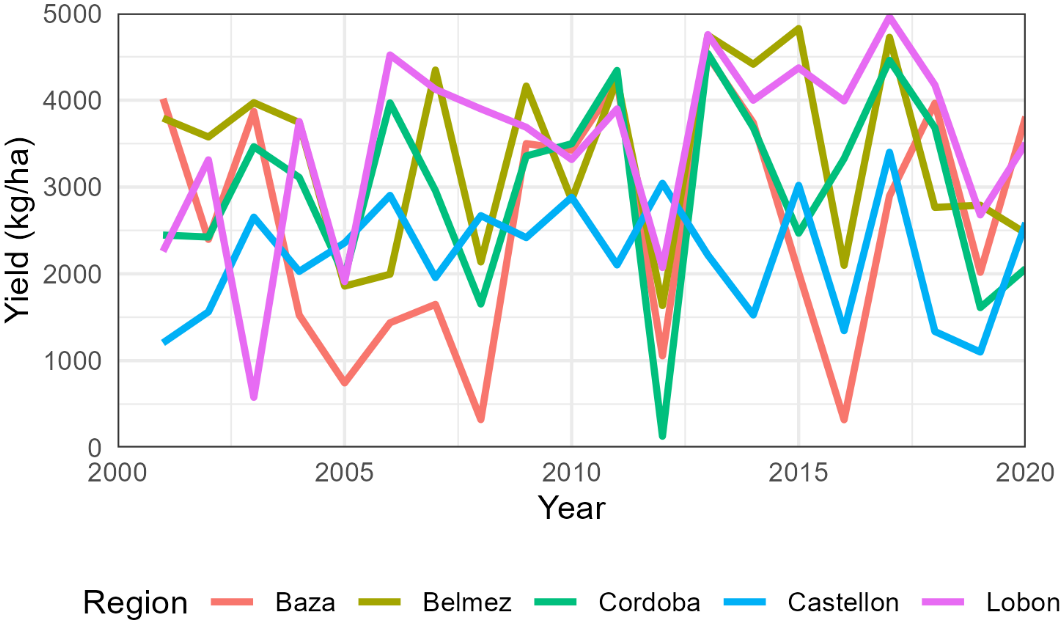
Average simulated wheat yields per region from 2001 to 2020. The cultivar change from SpringW1 to SpringW1+ occurred around 2010 but differed in each farm.

The total number of simulated years was 1240, with an average yield of 2945 kg/ha (**Table S4**). Per region, average wheat yields varied from 2215 kg/ha in Castellon to 3488 kg/ha in Lobon. Extreme values occurred in Cordoba in 2012 with only 130 kg/ha and Lobon in 2017 with 4959 kg/ha (**Figure 1**). The interannual coefficient of variation was maximum in Baza (54%) and minimum in Castellon and Lobon (31%) (**Table 5**).

**Table 5:** Statistics of yield (kg/ha) for wheat and sunflower in the period 2001-2020.

Wheat							
	Observed		Simulated				
	Cordoba	Granada	Lobon	Belmez	Cordoba	Baza	Castellon
Average	2546	1163	3488	3357	2957	2571	2215
SD	1077	398	1092	1078	1116	1384	691
CV	0.42	0.34	0.31	0.32	0.38	0.54	0.31

Sunflower							
	Observed		Simulated				
	Cordoba	Granada	Lobon	Belmez	Cordoba	Baza	Castellon
Average	1497	432	2441	817	1299	264	2345
SD	674	259	951	504	777	201	637
CV	0.45	0.60	0.39	0.62	0.60	0.76	0.27

Observed: Statistics for the provinces of Cordoba and Granada. Simulated: values obtained with crop models. Belmez and Cordoba are in the province of Cordoba while Baza is in the province of Granada (Lobon and Castellon are in neither).

The official statistics for the period 2001-2020 in the Cordoba province indicated an average yield of rain fed wheat of 2546 kg/ha (standard deviation SD 1077 kg/ha), The corresponding values in Granada were 1163 kg/ha (SD 398 kg/ha).

### 3.2 Sunflower yield

**Figure 2:**
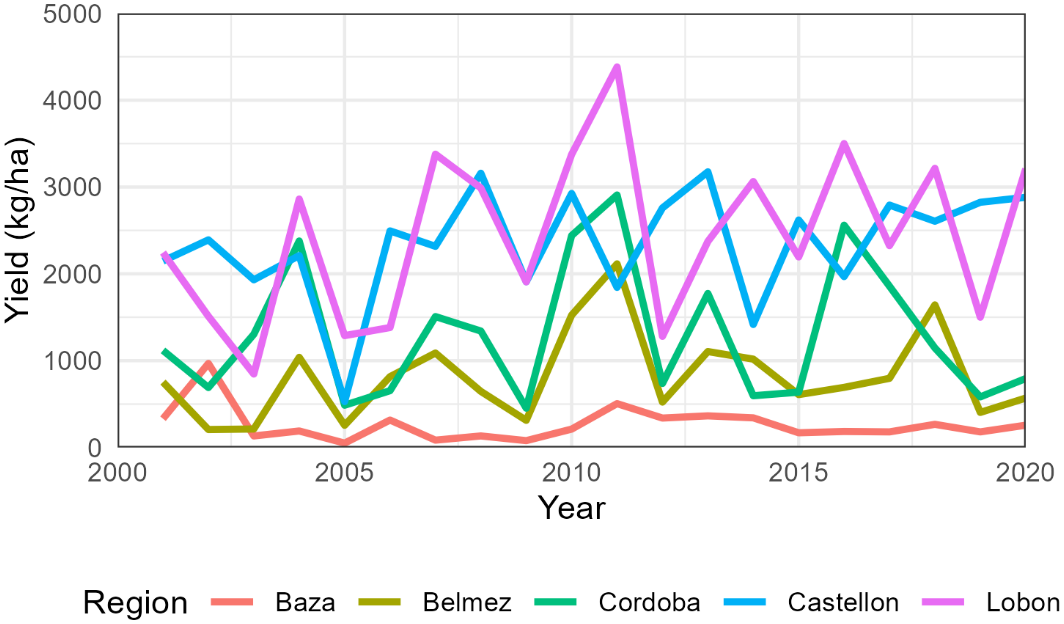
Average simulated sunflower yields for each region from 2001 to 2020. The cultivar change from AE353 to SW-101 occurred s around 2010 but differed in each farm.

The total number of simulated years was 1240, with an average yield of 1484 kg/ha (**Table S5**). Average regional sunflower yields were lowest in Baza (264 kg/ha) and highest in Lobon (2441 kg/ha). Extreme values occurred in Baza in 2005 with 51 kg/ha and Lobon in 2011 with 4382 kg/ha (**Figure 2**). The coefficient of variation was maximum in Baza (76%) and minimum in Castellon (27%) (**Table 5**).

Mean yield of sunflower according to the statistics was 1497 kg/ha (SD 674 kg/ha) in the Cordoba province and only 432 kg/ha (SD 259 kg/ha) in that of Granada (**Table 5**).

### 3.3 Wheat yield prediction (5 years training)

When the algorithms were trained on 5 years of wheat data (2010 – 2015), the best performing algorithm on the training dataset was ANN-12 (RMSE 4%) followed closely by ANN-9 and RF (RMSE 5% for both). The ranking for the test data was quite different as ANN-12 was the second worst algorithm (RMSE 141%) while RF was the best (RMSE 35%) followed by ANN-2, ANN-3, farm average and region average (RMSE 42%). The ranking was similar when other criteria were used with RF being the best in terms of MAE and second in maximum error, though it had a worse performance in terms of AICc (**Table 6**).

**Table 6:** Evaluation of alternative algorithms to predict wheat yield trained with 5 years of data (2011-2015) and tested on 5 years of data (2016-2020).

		Train				Test				
	Algorithm	RMSE	R2	k	ΔAICc	Bias	MAE	RMSE	ME	R2
**BASE**	**Avg (farm)**	37	23	124	1191	-12	35	42	111	7
	**Avg (region)**	38	18	10	901	-13	35	42	96	8
	***Lasso***	23	71	14	585	-56	61	69	137	-156
	***Ridge***	23	71	16	589	-56	61	69	137	-155
	**Linear**	23	71	16	588	-56	61	69	137	-156
	**Random forest**	5	99	184	308	-9	29	35	92	34
**ANN**	**ANN - 2**	13	90	29	298	-7	30	37	91	28
	**ANN - 3**	12	92	43	252	9	33	42	111	6
	**ANN - 4**	10	94	57	206	-32	43	50	120	-34
	**ANN - 5**	8	96	71	122	-108	110	134	279	-852
	**ANN - 7**	7	97	99	138	-215	220	256	497	-3389
	**ANN - 9**	5	99	127	0	-2	34	44	118	-2
	**ANN - 12**	4	99	169	300	-118	121	141	286	-958
**ANN with 50% dropout**	**ANN - 2+**	26	62	29	706	-26	38	45	98	-8
	**ANN - 3+**	22	72	43	650	-25	37	44	98	-3
	**ANN - 4+**	22	73	57	675	-31	41	48	97	-22
	**ANN - 5+**	20	78	71	654	-32	41	48	99	-23
	**ANN - 7+**	20	78	99	768	-35	43	50	104	-31
	**ANN - 9+**	17	83	127	816	-28	36	43	94	3
	**ANN - 12+**	16	85	169	1091	-34	42	48	118	-21

The base algorithms are the averages at the farm or region scale. RMSE: Root mean Square Error (%). R2: Coefficient of determination (%). Bias: Mean Bias Error (%). MAE: Mean Absolute Error (%). ME: Maximum Error in the data set (%). k: Number of non-zero coefficients. ΔAICc: Difference in AICc (with respect to lowest value). RMSE, MAE, Bias and ME have been standardized by dividing by the average yield in the corresponding dataset (training or testing). Numbers accompanying the Artificial Neural Networks (ANN’s) indicate the number of nodes in the hidden layer.

All ANN’s without dropout were better than RF in terms of AICc and the difference exceeded the threshold of 2 in all cases. The ranking of neural networks did not show any clear advantage from using larger networks or a dropout layer. Using the average yield of each farm to make predictions was a better approach than most of the ANN’s except when considering AICc as metric in the training data set (**Table 6**). Linear models showed a poorer performance than all other algorithms in training (RMSE 23% for Linear, *Ridge* and *Lasso*) and only some ANN’s in testing (RMSE 69%). The raw data for all simulations and algorithms can be found in **Table S6**.

### 3.4 Algorithms to predict sunflower yield (5 years training)

When the algorithms were trained on 5 years of sunflower data (2010 – 2015), the best performing algorithm on the training dataset was ANN-9 (RMSE 10%) followed by ANN-7 and RF (RMSE 12%). On the testing dataset the lowest MAE and RMSE was achieved by RF followed by average farm, while ANN-9 ranked 17^th^ out of 20 algorithms and linear models had an intermediate performance (**Table 7**). The best AICc was that of ANN-7, followed by ANN-9. The raw data and the predictions for all algorithms for this scenario are presented in **Table S7**.

**Table 7:** Evaluation of alternative algorithms to predict sunflower yield trained with 5 years of data (2011-2015) and tested on 5 years of data (2016-2020).

		Train				Test				
	Algorithm	RMSE	R2	k	ΔAICc	Bias	MAE	RMSE	ME	R2
**BASE**	**Avg (farm)**	47	61	124	896	2	30	42	179	69
	**Avg (region)**	52	53	10	651	1	32	42	159	68
	***Lasso***	40	73	14	482	53	55	64	170	26
	***Ridge***	40	73	16	492	53	55	65	171	25
	**Linear**	40	73	16	492	53	55	65	171	25
	**Random forest**	12	97	184	440	12	26	38	184	75
**ANN**	**ANN - 2**	22	91	29	168	7	33	45	199	64
	**ANN - 3**	18	95	43	56	-100	118	140	356	-254
	**ANN - 4**	18	94	57	111	121	122	141	284	-256
	**ANN - 5**	16	96	71	60	89	91	111	255	-121
	**ANN - 7**	12	98	99	0	10	56	75	305	0
	**ANN - 9**	10	98	127	35	-17	66	91	297	-47
	**ANN - 12**	25	90	169	911	39	44	60	185	36
**ANN with 50% dropout**	**ANN - 2+**	49	59	29	647	21	52	63	167	30
	**ANN - 3+**	45	65	43	641	26	60	73	187	5
	**ANN - 4+**	37	77	57	547	29	51	65	184	25
	**ANN - 5+**	35	78	71	572	31	51	65	182	24
	**ANN - 7+**	34	80	99	654	46	52	73	196	6
	**ANN - 9+**	28	86	127	678	26	40	56	180	44
	**ANN - 12+**	25	90	169	911	39	44	60	185	36

The base algorithms are the averages at the farm or region scale. RMSE: Root mean Square Error (%). R2: Coefficient of determination (%). Bias: Mean Bias Error (%). MAE: Mean Absolute Error (%). ME: Maximum Error in the data set (%). k: Number of non-zero coefficients. ΔAICc: Difference in AICc (with respect to lowest value). RMSE, MAE, Bias and ME have been standardized by dividing by the average yield in the corresponding dataset (training or testing). Numbers accompanying the Artificial Neural Networks (ANN’s) indicate the number of nodes in the hidden layer

Qualitatively, the patterns observed for predictions of sunflower and wheat yield were similar. Maximum errors were within 159% (region average) and 356% (ANN-3). The ranking of algorithms was similar for RMSE, MAE and R2 but quite different for maximum error and AICc (**Table 7**). Furthermore, the ranking differed between train and test data.

### 3.5 Effect of length of the training period

**Figure 3:**
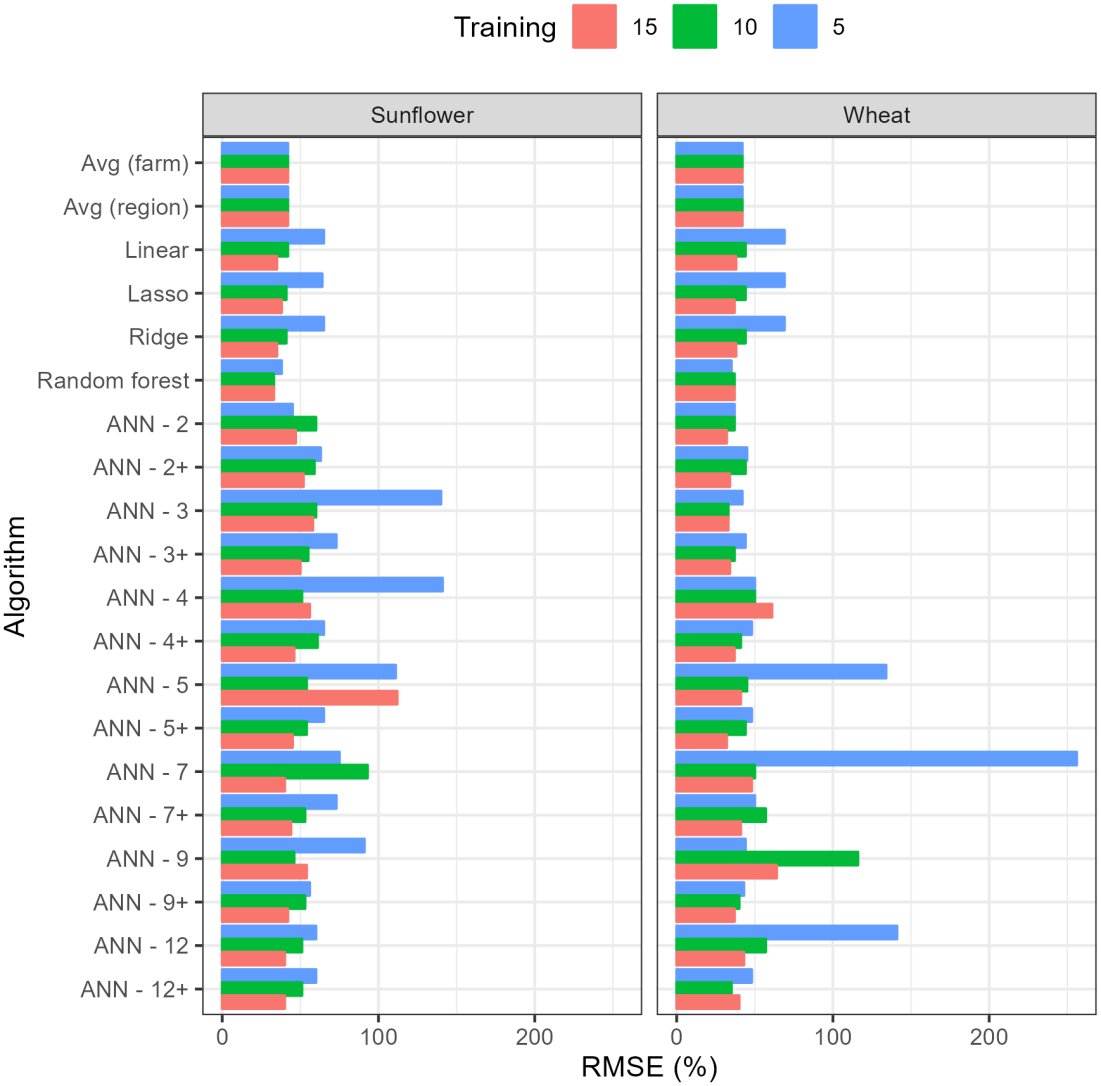
Root Mean Square Error (RMSE) on testing data of the different algorithms with training periods of 5, 10 and 15 years. RMSE has been standardized by dividing by the average yield in testing dataset. Numbers accompanying the Artificial Neural Networks (ANN’s) indicate the number of nodes in the hidden layer while the + sign indicates 50% dropout.

As the length of the period reserved for training increased from 5 years to 15 years, the RMSE on the testing dataset decreased for most algorithms (**Figure 3**). RF was the algorithm with the lowest RMSE for sunflower across all training duration and for wheat with 5 years of training. In the case of wheat with 10 and 15 years, ANN3 and ANN2 outperformed other algorithms, respectively. The full comparison of all predictive metrics for longer training periods (10 and 15 years) and both crops are provided in **Tables S8** and **S9**.

The reduction in RMSE for the best algorithm with the increase in training data was relatively small, considering the number of data points increased threefold. For wheat, it decreased from 35% (using RF) to 32% (using ANN-2) and for sunflower it decreased from 38% (using RF) to 33% (also RF). For other algorithms, the improvement in RMSE with training duration was more significant, as in the case of linear models where the decrease in RMSE allowed them to rank better among other algorithms (**Figure 3**). Within ANN’s, the results show that, as expected, 50% dropout reduced RMSE on the testing dataset, on average, by 20%, though ANN-2 and ANN-3 still performed better without dropout and the reduction in RMSE tended to be larger with larger neural networks. Unexpectedly, for most ANN’s, there was no clear pattern of variation between RMSE and the size of the neural network or the length of the training period, which may be a consequence of overfitting by the larger networks or the inability to converge to a global minimum during training.

### 3.6 Random partition of data for training and testing

When the training and testing dataset were sampled randomly from the original data regardless of the year (reserving 75% of the data for training, the remaining for testing), the best algorithm according to all criteria was RF for both wheat (RMSE 12%) and sunflower (RMSE 21%) (**Table 8**). Unlike in the previous section, the RMSE for random partitioning was almost the same for train (6-41%) and test (12-51%) and the ranking of algorithms was the same regardless of whether the error metrics were calculated on training or testing data. Furthermore, the lowest RMSE (6% for RF in wheat) was five times lower than the lowest RMSE using 15 contiguous years of training, (37%, **Table S9**) (despite using the same amount of data in both cases). Also, ANN-2, which was the best for 15 years of training on wheat (**Figure 3**; **Table S9**), performed significantly worse (RMSE 37%) than RF when using random partitioning (RMSE 25%).

**Table 8:** Evaluation of alternative algorithms of wheat and sunflower yield with random partition of training (75%) and test (25%) of data.

Wheat										
		Train				Test				
	Algorithm	RMSE	R2	k	ΔAICc	Bias	MAE	RMSE	ME	R2
**BASE**	**Avg (farm)**	37	24	124	2862	-1	35	42	119	4
	**Avg (region)**	38	19	10	2658	-1	32	39	104	19
	**Lasso**	32	41	15	2369	-1	27	33	92	41
	**Ridge**	32	41	16	2370	-1	27	33	92	41
	**Linear**	32	41	16	2370	-1	27	33	92	41
	**Random forest**	6	98	281	0	0	8	12	48	93
**ANN**	**ANN - 2**	24	68	29	1837	-1	20	25	84	66

Sunflower										
		Train				Test				
	Algorithm	RMSE	R2	k	ΔAICc	Bias	MAE	RMSE	ME	R2
**BASE**	**Avg (farm)**	46	65	124	2202	-1	38	53	206	56
	**Avg (region)**	51	58	10	2127	-1	37	51	200	59
	***Lasso***	41	73	16	1725	-1	32	42	170	72
	***Ridge***	41	73	16	1727	-1	32	42	170	72
	**Linear**	41	73	16	1724	-1	32	42	169	72
	**Random forest**	11	98	285	0	0	14	21	98	93
**ANN**	**ANN - 2**	36	79	29	1514	0	29	38	155	77

The baselines are the averages at the farm or region scale. RMSE: Root mean Square Error (%). R2: Coefficient of determination (%). Bias: Mean Bias Error (%). MAE: Mean Absolute Error (%). ME: Maximum Error in the data set (%). k: Number of non-zero coefficients. ΔAICc: Difference in AICc (with respect to lowest value). RMSE, MAE, Bias and ME have been standardized by dividing by the average yield in the corresponding dataset (training or testing). Numbers accompanying the Artificial Neural Networks (ANN’s) indicate the number of nodes in the hidden layer.

## 4 Discussion

We generated a dataset of simulated yields for wheat and sunflower using *DSSAT* which is a widespread crop simulation package. The crop models used respond to all the main environmental factors affecting crop growth and yield, including temperature, radiation, water and nitrogen availability, but cannot deal with other abiotic (micronutrients, salinity) or biotic (weeds, pests, diseases) limitations (Jones et al., 2003). Although randomness was used to generate the specific conditions at each farm or the exact year when the cultivar was changed, the simulation runs were deterministic. Furthermore, the simulations were performed independently for wheat and sunflower as two monocultures running in parallel. This is not realistic in agronomic practice but ensures that we can separate the effect of the crop simulation model (*Ceres-Wheat* or *OilcropSun*) on the analysis.

The simulated yields showed mean values and coefficients of variation close to the statistics for the provinces of Cordoba and Granada (**Table 5**). The simulated low values of sunflower in Baza (average 264 kg/ha) are fairly close to the provincial average (432 kg/ha) and are associated to the very low rainfall in the region (**Table 1**). This poor performance would probably lead actual farmers to stop growing sunflower, but this region was kept in the study to ensure a wide range of environmental conditions. On the other hand, Castellon was included as the inter-annual variation in rainfall differed significantly from the other regions, but sunflower and wheat are not actually grown in that area. Indeed, **Figures 1** and **2** show that average simulated yields from Castellon did not follow the same temporal pattern as the other four regions.

The algorithms were compared first using the last five years of the simulation (2016 – 2020) while different subsets of the earlier years were used for training. This emulates the situation where past data is used to extrapolate into the future, i.e., to forecast yields. Alternatively, there are situations when one wants to estimate yields (or any other variable) in the past by interpolating among known data points. This may be useful for filling gaps in time series, check self-declared yields by farmers or spatial interpolation when computing yields on a grid. In that case, a random partitioning of data for training and testing is adequate as long as no information leaks from the training to the testing dataset.

The results indicate that the type of data partitioning for testing will affect the overall predictive performance as well as the relative ranking of algorithms. For example, when 75% of the data was used for training, the resulting RMSE decreased by up to a factor of 5 depending on whether the training data was sampled randomly (**Table 8**, smaller RMSE, higher R2 on testing) or the training was done on the first 15 years of the time series (**Figure 3**; **Supplementary Table S9**, higher RMSE, lower R2 on testing). Only linear models (with or without regularization) performed similarly in both cases but they were never among the best algorithms. These large differences in performance illustrate the distinction between interpolation and extrapolation: using random partitioning to test an algorithm that is intended to forecast yields can overestimate significantly the actual performance of the algorithm during application. Indeed, the apparent success of ML in predicting yield in many studies (e.g. Khaki et al., 2020) may be the result of using random partitioning which should be restricted to applications where we need interpolation under past, well-known conditions.

By looking at the different scenarios, we conclude that RF appears to be most reliable and successful algorithm only beaten by the simplest ANN in two occasions though by a small margin (**Figure 3**). This is remarkable considering that none of the settings of the RF were tuned (the default settings were used), that it was very simple to set up (just one line of code in R using the package *ranger*) and fast to train (at the scale of seconds on a laptop). The only statistic where RF performed worse than was AICc, but it is important to understand that the degrees or freedom were estimated as the number of node splits (since there are no actual parameters in a RF), but this may be an overestimation, which would inflate AICc values. Despite all of these advantages, and extensive use in some fields of research (e.g., classification tasks in remote sensing, Belgiu and Drăguţ, 2016), RF is not often used for yield prediction (Jeong et al., 2016) but our results suggest that it would the best off-the-shelf algorithm for yield prediction.

One of the reasons why RF may perform better than ANN in this study is that the signal-to-noise ratio is low, especially when forecasting future yields, given that the best RMSE were still relatively high and the best R2 in the testing dataset did not exceed 0.8 (**Figure 3**, **Table S7**). It is known that a low signal-to-noise ratio and relatively small dataset would favor RF due to strong built-in regularization (Mentch and Zhou, 2020). Also, in machine learning competitions, algorithms based on decision trees often outcompete ANN when dealing with tabular data with relatively small sizes (e.g. reports from Kaggle competitions). In cropping systems, we have to cope with changes in management or the environment so it is not common to have yield series longer than 5 years for the same system. For instance, in the case of wheat the average duration of cultivars is around 7, with values lower than 5 in more developed agricultural systems (Brennan and Byerlee, 1991).

The poor performance of linear models did not change much with regularization. On the other hand, the dropout layer had a positive though insufficient effect on reducing predictive RMSE of ANN’s for wheat but not for sunflower. With 5 years of training, ANN-2 in wheat had lower RMSE than predictions using average farm yields from past data. This suggests that regularization methods may not fully compensate for excessive complexity leading to a trained model that slightly overfits the data. A further complication is that regularization based on penalties (*Lasso, Ridge*) or imposed sparsity (dropout layer) are dependent on specific settings (known as hyperparameters) which would have to be tuned for optimal performance. That tuning is generally performed *via* random partitioning of data, which would not necessarily improve predictions on extrapolation in the above. The tuning of hyperparameters also leads to a significant increase in computational requirements.

We have included the average yield at the farm or region level as a best-guess estimate in the absence of additional information from the farms. These estimates serve as the baseline for all the other algorithms. Unfortunately, very few published studies (Alvarez, 2009) report such a baseline. Furthermore, in some cases (e.g. Cai et al., 2019) only the coefficient of determination was used to select the best algorithm rather than looking at a wide range of error metrics. Even if some measure of predictive error is reported, without a proper baseline it will not be clear what is the gain in predictive power by using a machine learning algorithm (and collecting all the necessary additional data to train the algorithm). For example, for 5 years of training in wheat, the best algorithm (RF) had an RMSE of 0.35 compared to 0.42 when using the average yield of the farm during those years. For the same scenario, in sunflower, RF had an RMSE of 0.38 and predictions from farm average yield had an RMSE of 0.42.

Potential users should decide whether the cost of collecting additional input data and setting up the predictive framework is justified by the decrease in predictive error brought about by using a (regularized) linear model, RF or ANN. An analysis based on crop models, as illustrated in this study, could be used to justify such decisions, assuming that such models are available and reasonably well calibrated for the crop, cultivar and region of interest. Of course, such an analysis will always overestimate performance of predictive algorithms as long as weather variables are important for predictions (since in the real application the future weather would have to be predicted, introducing additional errors).

The need for faster empirical algorithms, able to consider many factors without explicit knowledge of the mechanisms behind, has led to the widespread adoption of ANN’s for yield prediction. In our virtual experiments, we have eliminated any additional source of error and selected directly the factors that are known to affect crop yield in both models. Furthermore, the number of datapoints available for training has been at least twice the number of parameters (except for ANN-12 and 5 years of training data, where the ratio was 1.8). Although there are cases where this ratio exceeds 1 (e.g. 6.2, Chapman et al., 2018) we can often find much lower values for the data/parameters ratio when ANN’s are being used (e.g. 0.12, Abrougui et al., 2019; see **Table S10** for a wider list of cases).

Despite our favorable setting, most ANN’s have performed worse than using average farm yields from past data. A possible explanation for the low performance of ANN’s may reside in the fact that crop yields may show discontinuous responses when data is aggregated at the seasonal level, which are hard to approximate by an ANN without large amounts of data. For instance, the level of stress at anthesis has a disproportionate effect on seed number in sunflower and wheat. This may be due to heat, frost or water stress occurring in just a few days (Pagani et al., 2017). If the data to train the algorithms is aggregated at the seasonal level, these critical responses will be missed. The same goes even for monthly (Khaki and Wang, 2019) or weekly (Khaki et al., 2020) data. Amplifying the number of inputs would not be the solution, as the number of parameters would become unmanageable because of the large data requirement (especially with ANN’s where the number of parameters scale rapidly with the number of features). Similar reasons may explain the poor performance of linear models and their regularized versions (*Ridge, Lasso*). On the other hand, RF may handle better the discontinuities by splitting the data recursively rather than approximating the underlying model with smooth functions.

The lack of predictive accuracy by mechanistic crop models is partly responsible for the use of empirical algorithms for prediction of future yields (Basso and Liu, 2019). A mixed approach, where mechanistic and empirical models are combined by expert analysts, as in the MARS unit of the Joint Research Centre (van der Velde et al., 2019), could lead to better results than either approach separately. In any case, we will always need biophysical crop models to explore uncharted conditions including not only global warming but also drastic changes in infrastructure or management (new irrigated areas, new cultivars) where experiments (and derived empirical models) are not possible (Flénet et al., 2008).

## 5 Conclusions

Machine learning algorithms showed a limited power (relative to a trivial average-yield baseline) for predicting yields of sunflower and wheat in different areas of Spain. Random partitioning of data for training and testing leads to underestimating model errors, as compared to time-dependent partition. Apart from being easier and faster to run, RF will always be at least as good as the best guess estimate (average of the farm yield on past data), a fact that is not guaranteed by ANN or linear models unless there is sufficient data.

## Data availability statement

The datasets presented in this study can be found in online repositories. The names of the repository/repositories and accession number(s) can be found below: https://www.uco.es/fitotecnia/tools/data_manuscript_morales_villalobos.zip.

## Author contributions

All authors listed have made a substantial, direct, and intellectual contribution to the work and approved it for publication.

## References

[B1] Abrougui K. Gabsi K. Mercatoris B. Khemis C. Amami R. Chahaibi S. (2019). Prediction of organic potato yield using tillage systems and soil properties by artificial neural network (ANN) and multiple linear regressions (MLR). Soil Tillage Res. 190, 202–208. doi: 10.1016/j.still.2019.01.011

[B2] Alvarez R. (2009). Predicting average regional yield and production of wheat in the Argentine pampas by an artificial neural network approach. Eur. J. Agron. 30, 70–77. doi: 10.1016/j.eja.2008.07.005

[B3] Bargoti S. Underwood J. P. (2017). Image segmentation for fruit detection and yield estimation in apple orchards. J. Field Robotics 34 (6), 1039–1060. doi: 10.1002/rob.21699

[B4] Basso B. Liu L. (2019). Chapter four - seasonal crop yield forecast: Methods, applications, and accuracies. Adv. Agron. 154, 201–255. doi: 10.1016/bs.agron.2018.11.002

[B5] Belgiu M. Drăguţ L. (2016). Random forest in remote sensing: A review of applications and future directions. ISPRS J. Photogrammetry Remote Sens. 114, 24–31. doi: 10.1016/j.isprsjprs.2016.01.011

[B6] Breiman L. (2001a). Statistical modeling: The two cultures (with comments and a rejoinder by the author). Stat. Sci. 16, 199–231. doi: 10.1214/ss/1009213726

[B7] Breiman L. (2001b). Random forests. Mach. Learn. 45, 5–32. doi: 10.1023/A:1010933404324

[B8] Breiman L. Friedman J. H. Olshen R. A. Stone C. J. (1984). Classification and regression trees. 1st ed (Milton Park, Abingdon, Oxfordshire, UK: Routledge).

[B9] Brennan J. P. Byerlee D. (1991). The rate of crop varietal replacement on farms: Measures and empirical results for wheat. Plant Var. Seeds 4, 99–106.

[B10] Cai Y. Guan K. Lobell D. Potgieter A. B. Wang S. Peng J.. (2019). Integrating satellite and climate data to predict wheat yield in Australia using machine learning approaches. Agric. For. Meteorology 274, 144–159. doi: 10.1016/j.agrformet.2019.03.010

[B11] Cao J. Zhang Z. Luo Y. Zhang L. Zhang J. Li Z.. (2021). Wheat yield predictions at a county and field scale with deep learning, machine learning, and google earth engine. Eur. J. Agron. 123, 126204. doi: 10.1016/j.eja.2020.126204

[B12] Chapman R. Cook S. Donough C. Li Lim Y. Vun Vui Ho P. Wai Lo K.. (2018). Using Bayesian networks to predict future yield functions with data from commercial oil palm plantations: A proof of concept analysis. Comput. Electron. Agric. 151, 338–348. doi: 10.1016/j.compag.2018.06.006

[B13] Dreyfus S. E. (1990). Artificial neural networks, back propagation, and the Kelley-bryson gradient procedure. J. guidance control dynamics 13 (5), 926–928. doi: 10.2514/3.25422

[B14] Flénet F. Debaeke P. Casadebaig P. (2008). Could a crop model be useful for improving sunflower crop management? Oléagineux Corps gras Lipides 15 (3), 158–161. doi: 10.1051/ocl.2008.0199

[B15] Hecht-Nielsen R. (1987). “Kolmogorov’s mapping neural network existence theorem,” in Paper presented at IEEE first international conference on neural networks(San Diego, CA, New York, NY, USA: IEEE Press), 1987.

[B16] Hoffman A. L. Kemanian A. R. Forest C. E. (2018). Analysis of climate signals in the crop yield record of sub-Saharan Africa. Global Change Biol. 24 (1), 143–157. doi: 10.1111/gcb.13901 28892592

[B17] Hornik K. Stinchcombe M. White H. (1989). Multilayer feedforward networks are universal approximators. Neural Networks 2 (5), 359–366. doi: 10.1016/0893-6080(89)90020-8

[B18] Iglesias A. (2006). “Use of DSSAT models for climate change impact assessment: Calibration and validation of CERES-wheat and CERES-maize in Spain,” in Climate variability, modelling tools and agricultural decision making. proc CGE hands-on training workshop on V&A assessment of the Asia and the pacific region (New York, NY: Nova Science Publishers), 20–24.

[B19] Jeong J. H. Resop J. P. Mueller N. D. Fleisher D. H. Yun K. Butler E. E.. (2016). Random forests for global and regional crop yield predictions. PloS One 11, e0156571. doi: 10.1371/journal.pone.0156571 27257967 PMC4892571

[B20] Johnson M. D. Hsieh W. W. Cannon A. J. Davidson A. Bédard F. (2016). Crop yield forecasting on the Canadian prairies by remotely sensed vegetation indices and machine learning methods. Agric. For. meteorology 218, 74–84. doi: 10.1016/j.agrformet.2015.11.003

[B21] Jones J. W. Hoogenboom G. Porter C. H. Boote K. J. Batchelor W. D. Hunt L. A.. (2003). *DSSAT* cropping system model. Eur. J. Agron. 18, 235–265. doi: 10.1016/S1161-0301(02)00107-7

[B22] Junta de Andalucia (2022) Avance de superficies y producciones. Available at: https://www.juntadeandalucia.es/organismos/agriculturaganaderiapescaydesarrollosostenible/servicios/estadistica-cartografia/estadisticas-agricolas/paginas/avance-superficie-producciones-agricolas.html (Accessed 10 July 2022).

[B23] Kaul M. Hill R. L. Walthall C. (2005). Artificial neural networks for corn and soybean yield prediction. Agric. Syst. 85 (1), 1–18. doi: 10.1016/j.agsy.2004.07.009

[B24] Khaki S. Wang L. (2019). Crop yield prediction using deep neural networks. Front. Plant Sci. 10, 621. doi: 10.3389/fpls.2019.00621 31191564 PMC6540942

[B25] Khaki S. Wang L. Archontoulis S. V. (2020). A CNN-RNN framework for crop yield prediction. Front. Plant Sci. 10, 1750. doi: 10.3389/fpls.2019.01750 32038699 PMC6993602

[B26] Kingma D. P. Ba L. J. (2014). Adam: A method for stochastic optimization. arXiv preprint arXiv:1412.6980.

[B27] Kira O. Nguy-Robertson A. L. Arkebauer T. J. Linker R. Gitelson A. A. (2016). Informative spectral bands for remote green *LAI* estimation in *C3* and *C4* crops. Agric. For. Meteorology 218, 243–249. doi: 10.1016/j.agrformet.2015.12.064

[B28] McCulloch W. S. Pitts W. H. (1943). A logical calculus of the ideas immanent in nervous activity. Bull. Math. Biophysics 7, 115–133. doi: 10.1007/BF02478259 2185863

[B29] Mentch L. Zhou S. (2020). Randomization as regularization: A degrees of freedom explanation for random forest success. J. Mach. Learn. Res. 21 (171), 1–36. doi: 10.48550/arXiv.1911.00190 34305477

[B30] Mohanty S. P. Hughes D. P. Salathé M. (2016). Using deep learning for image-based plant disease detection. Front. Plant Sci. 7, 1419. doi: 10.3389/fpls.2016.01419 27713752 PMC5032846

[B31] Noh H. K. Lu R. (2007). Hyperspectral laser-induced fluorescence imaging for assessing apple fruit quality. Postharvest Biol. Technol. 43 (2), 193–201. doi: 10.1016/j.postharvbio.2006.09.006

[B32] Pagani V. Guarneri T. Fumagalli D. Movedi E. Testi L. Klein T.. (2017). Improving cereal yield forecasts in Europe–the impact of weather extremes. Eur. J. Agron. 89, 97–106. doi: 10.1016/j.eja.2017.06.010

[B33] Ritchie J. T. Godwin D. C. Otter-Nacke S. (1985). CERES-wheat. a simulation model of wheat growth and development (College Station, TX: Texas A&M University College).

[B34] Srivastava N. Hinton G. Krizhevsky A. Sutskever I. Salakhutdinov R. (2014). Dropout: a simple way to prevent neural networks from overfitting. J. Mach. Learn. Res. 15 (1), 1929–1958. Available at: https://dl.acm.org/doi/10.5555/2627435.2670313.

[B35] Turing A. M. (1950). Computing machinery and intelligence. Mind 236, 433–460. doi: 10.1093/mind/LIX.236.433

[B36] van der Velde M. Biavetti I. El-Aydam M. Niemeyer S. Santini F. van den Berg M. (2019). Use and relevance of European union crop monitoring and yield forecasts. Agric. Syst. 168, 224–230. doi: 10.1016/j.agsy.2018.05.001

[B37] Villalobos F. J. Fereres E. (2016). Principles of agronomy for sustainable agriculture (NewYork, USA: Springer).

[B38] Villalobos F. J. Hall A. J. Ritchie J. T. Orgaz F. (1996). *Oilcrop-sun*: A development, growth and yield model of the sunflower crop. Agron. J. 88 (3), 403–415. doi: 10.2134/agronj1996.00021962008800030008x

[B39] Villalobos F. J. Sadras V. O. Soriano A. Fereres E. (1994). Planting density effects on dry matter partitioning and productivity of sunflower hybrids. Field Crops Res. 36, 1–11. doi: 10.1016/0378-4290(94)90047-7

[B40] Wang J. Xiao X. Bajgain R. Starks P. Steiner J. Doughty R. B.. (2019). Estimating leaf area index and aboveground biomass of grazing pastures using sentinel-1, sentinel-2 and landsat images. ISPRS J. Photogrammetry Remote Sens. 154, 189–201. doi: 10.1016/j.isprsjprs.2019.06.007

